# The prevalence and profile of spinal cord injury in public healthcare rehabilitation units in Gauteng, South Africa

**DOI:** 10.1038/s41394-023-00571-9

**Published:** 2023-04-14

**Authors:** Michael Alexandre Alves, Sonti Pilusa, Mokgadi Kholofelo Mashola

**Affiliations:** Department of Physiotherapy, School of Therapeutic Sciences, Faculty of Health Sciences, University of the Witwatersrand, Johannesburg, South Africa

**Keywords:** Epidemiology, Epidemiology

## Abstract

**Study design:**

Retrospective medical record review.

**Objective:**

To determine the prevalence and describe the profile of person with SCI (PWSCI) admitted in the public healthcare sector in Gauteng, South Africa.

**Setting:**

Specialized public healthcare rehabilitation units in Gauteng, South Africa.

**Methods:**

Medical records of PWSCI admitted to public healthcare rehabilitation units between 01 January 2018 and 31 December 2019 were perused. Data were collected anonymously and then summarised using descriptive and inferential statistics. Significance was set at *p* < 0.05.

**Results:**

386 of 998 participants (38.7%) were admitted following SCI and the mean age was 36.9 years. Most participants were male (69.9%), with females significantly more likely to sustain a NTSCI (*p* < 0.001), which was the least common cause of SCI (34.9%). Those sustaining a TSCI were found to be significantly younger than their NTSCI counterparts (*p* < 0.001). Assault was the leading cause of injury (35.2%), and a positive HIV status with the presence of comorbidities were found to be significant risk factors for developing a NTSCI (*p* < 0.001). Most injuries were between T7-T12 (39.9%) and were complete (56.9%). The rehabilitation length of stay 85.6 days, with a mortality rate of 6.48%.

**Conclusions:**

Gauteng has among the highest global proportion of TSCI due to assault. Of interest, more females sustained a NTSCI than their male counterparts. There is a need to strengthen SCI prevention strategies, particularly targeting assault in young males and infectious causes in females and older populations. Further epidemiological and outcomes-based research is required for PWSCI.

## Introduction

Spinal cord injury (SCI) is devastating, with morbidity not only limited to physical aspects but emotional, social, and vocational life areas too [1, 2]. Injury may occur as a result of trauma – referred to as traumatic SCI (TSCI) – or from disease or degeneration of the spinal cord – termed non-traumatic SCI (NTSCI) [3]. There are varying incidence rates of SCI worldwide with an estimated global incidence of TSCI of between 10.5 and 23 cases per 100,000 population [4, 5]. By region, estimates of NTSCI incidence range between 0.6 and 6.8 per 100 000 population [6]. Falls are fast becoming the leading cause of TSCI in high-income regions worldwide with high incidences of tumours and degenerative conditions in those with NTSCI [5–7]. In low and middle-income regions, transportation-related injuries remain the leading cause of TSCI while high incidences of infection-related injury remains prominent in those with NTSCI [8]. Males, those between the age of 15 and 29 as well as those above 65 years are at highest risk of TSCI while a proportionate rise in age is associated with an increased risk of NTSCI [9].

Statistics South Africa uses the term ‘disability’ as an umbrella term to refer to conditions affecting vision, hearing, communication, mobility, cognition and self-care [10]. In South Africa, SCI surveillance data is scanty with SCI epidemiological data predominantly based in two provinces, the Western Cape [11–14] and KwaZulu-Natal [15, 16]. The country – Gauteng Province in particular – continues to report the highest proportion of assault-related TSCI worldwide with the NTSCI population suffering largely from HIV- and tuberculosis-related afflictions [5, 16]. A wide range of incidence has been published with estimates of between 20.0 and 123 injuries per million population [13, 16] with no actual prevalence rates published in Africa.

Rehabilitation of persons with SCI (PWSCI) is necessary to improve the individual’s ability to perform functional activities and empower them and their families with the knowledge and skills to prevent secondary health complications [17]. Admission to a specialised SCI unit for acute and/or rehabilitative care has been associated with a shortened length of stay, fewer health complications and lower mortality rates [18–20]. To support the global agenda of strengthening rehabilitation services worldwide, part of the “Rehabilitation 2030” initiative aims to collect information relevant to rehabilitation care [21]. Gauteng Province, housing the country’s executive capital city and being the most populous province [22], lacks recent and thorough data with current research outdated and limited [23–25]. Without adequate epidemiological data, planning for SCI care from an acute to chronic setting becomes challenging [26, 27]. This study thus aims to update and describe the profile of PWSCI receiving rehabilitation in the public healthcare sector in Gauteng, South Africa.

## Methods

### Study design

This quantitative study used a retrospective, medical record review study approach. Convenience sampling was used to select medical records appropriately fitting the inclusion criteria.

### Study setting and population

This study was conducted at five specialised rehabilitation units for PWSCI in the public healthcare sector of Gauteng Province. All medical records for adult individuals (≥18 years) who had sustained a confirmed SCI, irrespective of cause of injury, were considered for this study. The medical records were deemed eligible if the individual had been admitted to any of the five specialised SCI units in the public healthcare sector in Gauteng between the 1^st^ of January 2018 and the 31^st^ of December 2019. Those with no neurological fallout following SCI (classified as “E” according to the American Spinal Injury Association Impairment Scale) and those with neurological fallout as a result of cranial and/or peripheral nerve system pathology were excluded from the study. No sample size was calculated for this study as all medical records that met the inclusion criteria were included in the study.

### Data collection

A pilot study of ten medical records was conducted to ensure practicality, familiarity, efficiency and quality of the data collection form and overall study procedure [28]. No amendments needed to be made to the data collection form or to the study in its entirety. Data was collected using a specifically designed data collection form which included all aspects of the International Spinal Cord Injury Core Data Set (ISCICDS) [29, 30] as well as the following data: HIV status, presence and type of comorbidities, occupation, marital status, the highest level of education, the place from where the participant was admitted into rehabilitation, rehabilitation mortality as well as the type of mobility aid used at discharge from rehabilitation. Rehabilitation ward records were used to identify patients admitted into the rehabilitation ward/s across the study period with these records being pursued and those fitting the inclusion criteria enroled in the study.

### Data analysis

Data were summarised using descriptive statistics with inferential statistics used to investigate relationships between independent and dependent variables. The SPSS v27 was used to analyse the data and continuous variables were analysed using mean with standard deviation as well as median and interquartile ranges. Categorical variables were analysed using frequencies and percentages. Only data that was available and collectable in the medical records were included in the analysis. Our data were not normally distributed, as confirmed by a less than 0.05 significance of the Shapiro-Wilk test of normality. Hence, non-parametric tests were used to analyse the data. The Mann–Whitney U test was used to determine whether significant differences existed between continuous variables of age, days to admission, length of, stay and cause of injury. The Chi-square measure of association and the Fischer’s exact test were used to determine the associations between independent and dependent categorical variables. The Spearman correlation coefficient was used to determine the relationship between age, days to admission and length of stay. Univariate and multivariate binary logistic regression was used to determine the predictors of cause of injury. Significance was set at *p* < 0.05 with a 95% confidence interval.

### Ethical approval

Ethical clearance was granted by the University of the Witwatersrand’s Human Research Ethics Committee (certificate number M200582). Additionally, permission was granted by the management of each rehabilitation unit. The medical records and data collection sheet remain anonymous and this paper does not disclose any personal information.

## Results

During the two-year study period, 998 patients were admitted for rehabilitation to Gauteng’s five specialised rehabilitation units. Of these, 386 had sustained a SCI and met all the inclusion criteria and were thus included in the study.

### Sociodemographic profile

The mean (SD) age of study participants was 36.90 (12.12) years while the median age was 34 years (range 18–75). The most common age group was 31–45 (*n* = 148, 38.85%), closely followed by those 18–30 (*n* = 147, 38.58%) and then those 46–60 (*n* = 66, 17.32%) and 61 and over (*n* = 20, 5.25%). Participants with TSCI were significantly younger than those with NTSCI (*p* < 0.001). Males were more commonly injured than females (*n* = 270, 69.95%) across the two-year study period. Of note, females were more commonly affected by NTSCI than their male counterparts, with a male to female ratio of 5.44:1 and 0.75:1 for TSCI and NTSCI respectively. A positive and significant association was shown to exist between males and TSCI as well as between females and NTSCI (*p* < 0.001). Most of the participants in this study were Black (*n* = 357, 92.49%) and resided in the City of Tshwane (*n* = 91, 24.53%). A significant association was found to exist between formal employment and TSCI, with approximately half of the participants formally employed at the time of admission (*n* = 161, 48.20%) (*p* < 0.001). Unemployment and NTSCI were also found to be significantly associated (*p* < 0.001). A third of study participants had a matric certificate (*n* = 89, 35.60%). Most of the participants were never married (*n* = 226, 65.89%), with a significant association between TSCI and never being married, as well as between NTSCI and being married (*p* < 0.001). The sociodemographic profile of the included participants is presented in Table 1.

**Table 1:** Sociodemographic Profile.

	Overall (*n* = 386)	TSCI (*n* = 251)	NTSCI (*n* = 131)	*p*-value
Age				0.00^a^
Mean (SD)	36.90 (12.12)			
Median	34	31	44	
Range	18–75			
Q1–Q3		26–39.75	32.5–51.5	
Gender, *n* (%)	386	251	135	0.00^a^
Male	270 (69.95)	212 (84.46)	58 (42.96)	
Female	116 (30.05)	39 (15.54)	77 (57.04)	
Ethnicity, *n* (%)	386	251	135	0.08^b^
Black	357 (92.49)	236 (94.02)	121 (89.63)	
White	13 (3.37)	5 (2.00)	8 (5.93)	
Coloured	14 (3.63)	10 (3.98)	4 (2.96)	
Indian	2 (0.52)	0	2 (1.48)	
Area of residence, *n* (%)	371	245	126	0.06^b^
City of Tshwane	91 (24.53)	51 (20.82)	40 (31.75)	
City of Johannesburg	88 (23.72)	62 (25.31)	26 (20.63)	
City of Ekurhuleni	76 (20.49)	58 (23.67)	18 (14.29)	
West Rand	15 (4.04)	7 (2.86)	6 (4.76)	
Sedibeng	13 (3.50)	12 (4.90)	3 (2.38)	
Another Province	88 (23.72)	55 (22.45)	33 (26.19)	
Occupation, *n* (%)	334	229	105	0.00^a^
Unemployed	90 (26.95)	49 (21.39)	41 (39.05)	
Self-employed	53 (15.87)	39 (17.03)	14 (13.33)	
Formally employed	161 (48.20)	124 (54.15)	37 (35.24)	
Student	17 (5.09)	14 (6.11)	3 (2.86)	
Pensioner	13 (3.89)	3 (1.31)	10 (9.52)	
Marital Status, *n* (%)	343	226	117	0.00^a^
Never Married	226 (65.89)	171 (75.66)	55 (47.01)	
Married	93 (27.11)	45 (19.91)	48 (41.03)	
Divorced	11 (3.21)	6 (2.65)	5 (4.27)	
Widowed	13 (3.79)	4 (1.77)	9 (7.69)	
Education, *n* (%)	250	177	73	0.22
Grade 9 or lower	59 (23.60)	47 (26.55)	12 (16.67)	
Grade 10–11	84 (33.60)	61 (34.46)	23 (31.94)	
Grade 12	89 (35.60)	59 (33.33)	30 (41.67)	
Post-secondary	18 (7.20)	10 (5.65)	8 (11.11)	

Young, Black males are the most prevalent population group admitted for rehabilitation after TSCI. In the NTSCI population group, older women were significantly more common. A relatively similar proportion of participants resided in the City of Tshwane, City of Johannesburg as well as in The City of Ekurhuleni at the time of their injury. Most participants were formally employed and had never married at their time of injury with significant associations existing between these sociodemographic factors and injury aetiology. No association was found between participants’ highest level of education and injury aetiology.

^a^Significant *p* value.

^b^Fischer’s exact test.

### Injury profile

**Fig. 1:**
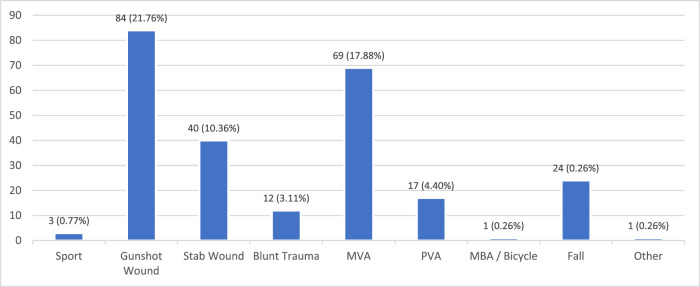
Aetiology of traumatic spinal cord injury. Traumatic SCI accounted for almost two-thirds of injury with injuries mostly resulting from gunshots and motor vehicle accidents.

**Fig. 2:**
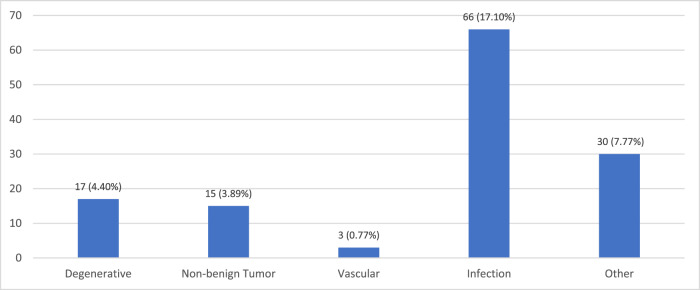
Aetiology of non-traumatic spinal cord injury. Infection-related causes were the most common followed by other NTSCI causes (most commonly cancer) and degenerative conditions.

The overall prevalence of SCI in Gauteng public healthcare rehabilitation units was 38.67%. Traumatic SCI (*n* = 251, 65.03%) was more common than NTSCI (*n* = 135, 34.97%). The most common cause of injury overall was as a result of assault (*n* = 136, 35.23%) followed by transportation injuries (*n* = 87, 22.54%) and infectious causes (*n* = 66, 17.10%) – most commonly HIV- and tuberculosis-related. The injury frequency for TSCI and NTSCI is outlined below in Figs. 1 and 2 respectively. Of note, 5.18% of all participants (*n* = 20) were diagnosed with cancer and were included in the “other NTSCI” group.

Most of the participants had complete lesions and were classified as AIS A as per the International Standards for Neurological Classification of Spinal Cord Injury (ISNCSCI) (*n* = 170, 56.86%). Traumatic SCI was found to be significantly associated with an AIS A injury (*p* < 0.001), while NTSCI and motor incomplete lesions (AIS C and AIS D) were significantly associated (*p* < 0.001). The most common site of injury was the lower thoracic region of T7-T12 (*n* = 105, 39.92%) (Table 2).

**Table 2:** Injury severity and classification.

	Overall	TSCI	NTSCI	*p*-value
Injury classification, *n* (%)	299	232	67	0.00^a^
AIS A	170 (56.86)	143 (61.64)	27 (40.30)	
AIS B	15 (5.02)	12 (5.17)	3 (4.48)	
AIS C	65 (21.74)	38 (16.38)	27 (40.30)	
AIS D	16 (5.35)	10 (4.31)	6 (8.96)	
Incomplete syndrome	33 (11.04)	29 (12.50)	4 (5.97)	
Level of injury, *n* (%)	263	197	66	0.88
C1–C4	22 (8.37)	15 (7.6)	7 (10.3)	
C5–C8	33 (12.55)	26 (13.2)	7 (10.3)	
T1–T6	70 (26.62)	51 (25.9)	19 (27.9)	
T7–T12	105 (39.92)	80 (40.6)	25 (36.8)	
L1–L5	35 (13.31)	25 (12.7)	10 (14.7)	

The majority of the participants sustained a complete, AIS A injury as per the International Standards for Neurological Classification of SCI (ISNCSCI). Participants with NTSCI were significantly more likely to sustain an incomplete injury compared to their TSCI counterparts. The lower thoracic spine (T7–T12) was the area most commonly injured, followed by the upper thoracic spine (T1–T6).

^a^Significant *p* value.

Most participants in this study were admitted into rehabilitation from tertiary-level healthcare institutions (*n* = 223, 62.82%). There was a mean (SD) time of 64.48 (59.78) days between the day of injury and day to rehabilitation admission (median 46 days, range 1–369), with the NTSCI group having a significantly longer waiting period than their TSCI counterparts (*p* < 0.001). Once admitted, the mean (SD) length of stay in rehabilitation was 85.59 (63.21) days (median 72 days, range 5–388 days). Most of the study participants were classified as HIV negative (*n* = 151, 69.91%) and most did not present with any health comorbidity (*n* = 243, 73.19%).

Significant associations were found to exist between the NTSCI group and being HIV positive (*p* < 0.001) as well as having a health comorbidity (*p* < 0.001). The most common comorbidity was tuberculosis (*n* = 44, 11.40%) followed by hypertension (*n* = 26, 6.74%) and diabetes (*n* = 5, 1.30%). Vertebral fractures were common (*n* = 256, 77.11%), with the high proportion in the NTSCI group likely a reflection of the disease process associated with spinal tuberculosis. Just under one half of the participants received spinal surgery (*n* = 154, 44.51%). There was a significant association between the TSCI group and both of these data sets (*p* < 0.001). Associated injuries (as defined by the ISCICDS and only recorded in persons with TSCI) were found to exist in just under half of the participants in the TSCI group (*n* = 85, 43.82%). The injury profile of the included participants is presented in Table 3.

**Table 3:** Injury profile.

	Overall	TSCI	NTSCI	*p*-value
Source of referral				0.57
Tertiary hospital	223 (62.82)	140 (58.82)	83 (71.55)	
Regional hospital	74 (20.85)	60 (25.21)	14 (12.07)	
District hospital	40 (11.27)	28 (11.76)	12 (10.34)	
Home	10 (2.82)	6 (2.52)	4 (3.45)	
Other	7 (1.97)	4 (1.68)	3 (2.59)	
Time to admission				0.00^a^
Mean (SD)	64.48 (59.78)	60.39 (53.98)	90.56 (72.02)	
Median	46	43	79	
Range	1–369			
Q1–Q3		25–76	44.25–116.5	
Length of stay				0.06
Mean (SD)	85.59 (63.21)	88.96 (63.57)	77.91 (61.73)	
Median	72	73	66	
Range	5–388			
Q1–Q3		46–113	29–109	
HIV status, *n* (%)	216	133	83	0.00^a^
Positive	65 (30.09)	21 (15.79)	44 (53.01)	
Negative	151 (69.91)	112 (84.21)	39 (46.99)	
Comorbidities, *n* (%)	332	219	113	0.00^a^
Yes	89 (26.81)	20 (9.13)	69 (61.06)	
No	243 (73.19)	199 (90.87)	44 (38.94)	
Vertebral fracture, *n* (%)	332	226	106	0.00^a^
Yes	256 (77.11)	211 (93.4)	45 (42.5)	
No	76 (22.89)	15 (6.6)	61 (57.5)	
Spinal surgery, *n* (%)	346	230	116	0.00^a^
Yes	154 (44.51)	141 (61.30)	51 (43.97)	
No	192 (55.49)	89 (38.70)	65 (56.03)	
Associated injury, *n* (%)	307	194	113	0.00^a,b^
Yes	85 (27.69)	85 (43.82)	N/A	
No	222 (72.31)	109 (56.28)	113	

Approximately two-thirds of rehabilitation transfers were conducted from tertiary-level healthcare institutions. Those sustaining NTSCI had a significantly longer time to admission with no association identified between aetiology and length of rehabilitation stay. A positive HIV status as well as the presence of comorbidities were significant factors in the acquisition of a NTSCI. Three-quarters of study participants had a vertebral fracture whilst one-quarter had an associated injury. Approximately half of all participants underwent spinal surgery.

^a^Significant *p* value.

^b^Fischer’s exact test.

Overall, most of the participants in this study were discharged from rehabilitation (*n* = 342, 88.60%) with an overwhelming proportion being discharged to a private residence (*n* = 330, 97.29%). Successful discharge from rehabilitation was significantly associated with the TSCI group with those sustaining a NTSCI more likely to not be discharged (*p* < 0.001). Of those that were not discharged from rehabilitation, 25 participants demised (6.48%), eight refused further rehabilitation care (2.07%), seven absconded (1.81%), and four became medically unstable and were deemed unfit to continue rehabilitation and were transferred back to acute care (1.04%). No participants made use of a ventilator or supplementary oxygen at discharge. Manually-operated wheelchairs (*n* = 185, 57.81%) were the most common type of assistive device used for mobility at discharge from rehabilitation. The injury profile information related to rehabilitation completion is provided in Table 4.

**Table 4:** Injury profile information related to rehabilitation completion/termination.

	Overall	TSCI	NTSCI	*p*-value
Completed rehabilitation (%)	386	251	135	0.00^a^
Yes	342 (88.60)	232 (92.43)	110 (81.48)	
No	44 (11.40)	19 (7.57)	25 (18.52)	
Discharge destination, *n* (%)	338	231	107	0.00^a,b^
Private residence	330 (97.92)	227 (98.27)	103 (96.26)	
Another hospital	7 (2.08)	4 (1.73)	3 (2.80)	
Assisted-living residence	1 (0.30)	0	1 (0.93)	
Mobility aid used at discharge, *n* (%)	320	221	99	0.41
Battery-operated WC	34 (10.63)	26 (11.76)	8 (8.01)	
Manually-operated WC	185 (57.81)	132 (59.73)	53 (53.54)	
Walking frame/Rollator	50 (15.63)	30 (13.57)	20 (20.20)	
Elbow crutches/Cane/Stick	23 (7.19)	14 (6.33)	9 (9.09)	
No mobility aid	28 (8.75)	19 (8.60)	9 (9.09)	

Most study participants reported successful completion of rehabilitation with almost all participants discharged home. A notable proportion of those who did not complete rehabilitation successfully had a NTSCI; were discharged to another hospital or to an assisted-living residence. Most participants made use of a manually-operated wheelchair at completion of rehabilitation.

^a^Significant *p* value.

^b^Fischer’s exact test.

Binary logistic regression was used to explore sociodemographic predictors of SCI (Table 5). The probability of acquiring a TSCI was found to increase by 0.94 for every one-unit increase in age (*p* < 0.001) and by 0.14 in females as compared with their male counterparts (*p* < 0.001). Being unemployed was found to increase the odds of acquiring a TSCI by 0.37 (*p* < 0.001).

**Table 5:** Sociodemographic predictors of traumatic spinal cord injury.

		Univariate		Multivariate	
Predictor		EXP(B)	*p*-value	EXP(B)	*p*-value
		[95% CI]		[95% CI]	
Age		0.93 [0.91–0.95]	0	0.94 [0.92–0.96]	**0.00**
Sex	Male	Reference	Reference	Reference	Reference
	Female	0.13 [0.08–0.22]	0	0.14 [0.09–0.24]	**0.00**
Occupation	Formally Employed	Reference	Reference	Reference	Reference
	Self Employed	0.83 [0.41–1.70]	0.61	0.75 [0.34–1.65]	0.47
	Unemployed	0.37 [0.21–0.64]	0	0.37 [0.20–0.72]	**0.00**
	Student	1.39 [0.38–5.11]	0.62	1.08 [0.25–4.72]	0.92
	Pensioner	0.09 [0.02–0.34]	0	0.51 [0.10–2.55]	0.41

Age, sex and being unemployed were all significant predictors of sustaining a TSCI. The probability of acquiring a TSCI increased by 0.94, 0.14 and 0.37 for every one-unit increase in age, gender and unemployment respectively.

Values in bold indicate significant values.

Traumatic SCI was found to be a significant predictor for sustaining a vertebral fracture as well as for requiring spinal surgery (*p* < 0.001) as illustrated in Table 6.

**Table 6:** Predictors relating to the injury profile.

Injury profile factor		Univariate		Adjusted for age and sex	
	Aetiology	EXP(B)	*p*-value	EXP(B)	*p*-value
		[95% CI]		[95% CI]	
Vertebral Fracture	NTSCI	Reference	Reference	Reference	Reference
	TSCI	0.05 [0.03–0.10	0	0.04 [0.02–0.08]	**0.00**
Mortality	NTSCI	Reference	Reference	Reference	Reference
	TSCI	0.27[0.12–0.62]	0	0.60[0.22–1.61]	0.31
Spinal Surgery	NTSCI	Reference	Reference	Reference	Reference
	TSCI	2.03 [1.29–3.20]	0	0.51 [0.30–0.87]	**0.01**
Mobility Aid Used at Discharge					
Wheelchair	NTSCI	Reference	Reference	Reference	Reference
	TSCI	0.96[0.41–2.29]	0.93	2.13[0.78–5.83]	0.14
Walking Frame/Rollator	NTSCI	Reference	Reference	Reference	Reference
	TSCI	0.60[0.22–1.63]	0.32	1.31[0.41–4.19]	0.65
Crutches/Cane/Walking Stick	NTSCI	Reference	Reference	Reference	Reference
	TSCI	0.70[0.21–2.31]	0.56	1.07[0.27–4.29]	0.92

Traumatic SCI was a significant predictor for the presence of a vertebral fracture and undergoing spinal surgery.

Values in bold indicate significant values.

Binary logistic regression was also used to explore sociodemographic factors in relation to injury outcomes (Table 7). The probability of sustaining a vertebral fracture was found to increase significantly by 0.37 in females and 0.26 in the unemployed respectively (*p* < 0.001). A significant correlation exists between rehabilitation mortality and age with the risk of death increasing by 0.92 for every one-unit increase in age (*p* < 0.001).

**Table 7:** Sociodemographic predictors of rehabilitation outcomes.

Predictor		33.2 Univariate		Multivariate	
		43.2 EXP(B)	*p*-value	EXP(B)	*p*-value
		[95% CI]		[95% CI]	
Vertebral fracture	Age	0.98 [0.96–1.00]	0.06	0.99 [0.96-1.02]	0.54
	Sex				
	Male	Reference	Reference	Reference	Reference
	Female	0.32 [0.19–0.55]	0	0.37 [0.20–0.70]	**0.00**
	Occupation				
	Formally employed	Reference	Reference	Reference	Reference
	Self employed	1.21 [0.46–3.17]	0.7	1.15 [0.43–3.07]	0.78
	Unemployed	0.23 [0.12–0.44]	0	0.26 [0.14–0.50]	**0.00**
	Student	2.72 [0.34–21.58]	0.35	3.57 [0.41–30.88]	0.25
	Pensioner	0.27 [0.07–1.04]	0.06	0.44 [0.09–2.07]	0.3
Spinal surgery	Age	1.02 [1.00–1.03]	0.1	1.02 [1.00–1.05]	0.06
	Sex				
	Male	Reference	Reference	Reference	Reference
	Female	1.33 [0.84–2.12]	0.23	1.12 [0.65–1.92]	0.68
	Occupation				
	Formally employed	Reference	Reference	Reference	Reference
	Self employed	0.78 [0.41–1.51]	0.46	0.76 [0.39–1.48]	0.42
	Unemployed	1.02 [0.60–1.74]	0.94	0.99 [0.57–1.73]	0.98
	Student	1.85 [0.63–5.46]	0.26	2.42 [0.76–7.69]	0.13
	Pensioner	0.53 [0.13–2.13]	0.37	0.29 [0.06–1.31]	0.11
Mortality	Age	0.92 [0.89–0.95]	0	0.92 [0.88–0.97]	**0.00**
	Sex				
	Male	Reference	Reference	Reference	Reference
	Female	0.62 [0.27–1.43]	0.27	1.27 [0.31–5.23]	0.74
	Occupation				
	Formally employed	Reference	Reference	Reference	Reference
	Self employed	0.97 [0.22–3.41]	0.84	0.94 [0.23–3.79]	0.93
	Unemployed	4.65 [0.57–37.82]	0.15	4.29[0.51–36.41]	0.18

The probability of sustaining a vertebral fracture increased significantly by 0.37 and 0.26 in females and those who were unemployed respectively. A significant correlation exists between age and mortality with the risk of death increasing by 0.92 for every one-unit increase in age.

Values in bold indicate significant values.

## Discussion

To our knowledge, this is the first study to report on the prevalence and profile of SCI in each of Gauteng’s public healthcare rehabilitation units. Across our two-year study period, 386 PWSCI were admitted to Gauteng’s five public rehabilitation unit’s – an average of 193 admissions per year. Hart [24] reported that there were 1 203 admissions to a single public healthcare rehabilitation unit in Gauteng over an 11-year period between 1988 and 1998 – an average of 109 admissions per year. In the country’s acute-care units, the number of admissions averaged between 13 and 349 admissions per year [13, 14]. The contrasting study settings, levels of care and time periods makes comparison difficult, highlighting the need for ongoing SCI surveillance in a public, private, acute and rehabilitative setting.

Young males continue to be significantly most at risk of sustaining a TSCI, with world-high levels of assault – largely due to gunshot and stab wounds – most prominent (*p* < 0.001). These findings are consistent with literature previously published in Gauteng [23, 24] the Western Cape [11] and Kwazulu-Natal [16]. Crime levels, particularly those related to assault, have been shown to be highest in Gauteng [31]. Disabling injuries contribute significantly to the national burden of disease putting a strain on an already burdened healthcare system [32]. Interventions to address the prevention of crime, particularly in the male population, must be strengthened.

Notable findings were discovered in the NTSCI population. We found that significantly more females sustained a NTSCI (*p* < 0.001) with this population being significantly older too (*p* < 0.001). This reflects previous literature from Kwazulu-Natal which, to the authors knowledge, is the only literature worldwide where more females are affected by NTSCI than males [15, 16]. This may be a direct reflection and association between the high proportion of infectious-related NTSCI and a country with one of the highest prevalence’s of HIV and tuberculosis in Sub-Saharan Africa, particularly in women [33]. These conditions are associated with serious long-term health implications and, superimposed on a NTSCI, may talk to the significant rate of unemployment in this study group (*p* < 0.001) [31, 33, 34]. It is imperative that the country urgently addresses the prevention and management of HIV at various healthcare levels in line with the “90-90-90” programme as outlined by the Joint United Nations Programme on HIV/AIDS [35]. We also noted that those with a NTSCI waited significantly longer for rehabilitation admission (*p* < 0.001). This may be a reflection of the usually gradual and progressive nature of onset of most NTSCI conditions. Further, one must note that medical work-ups and investigations may be delayed in a heavily over-burdened and under-funded public healthcare sector. A significant proportion of participants in the NTSCI group did not complete their rehabilitation stay (*p* < 0.001). This may be because of the older age and direct correlation to a higher mortality rate as well as the higher proportion of HIV and other health comorbidities of this group potentially affecting their medical stability. We suggest that more research is conducted in the NTSCI populations, particularly with regards to disability and functional outcomes.

Mixed results with regards to injury classification and severity were found as compared with previously published South African literature. Complete injury and paraplegia was found to be most prevalent in our study, similar to outdated Gauteng literature by Hart and Williams [23] and Hart [24]. This differs to published research from Kwazulu-Natal [16] and the Western Cape [11–14] where incomplete injuries and quadriplegia were most common. Study setting and injury aetiology is likely to have influenced these findings. Gauteng’s literature focussed on the rehabilitative stage and reflected high levels of SCI as a result of assault while Kwazulu-Natal and the Western Cape reflected findings from the acute stage with high levels of transportation-related injuries. Those with injuries as a result of transportation may also be compensated by the country’s Road Accident Fund and have received their rehabilitation in the private healthcare sector. Patients with complete injuries experience more functional fallout, are at higher risk of secondary complications and have a higher risk of mortality post discharge [36–38]. There is a need to strengthen access to rehabilitation care with focussed and appropriate planning of rehabilitation services minimising poor health outcomes in those with SCI.

Our study investigated associations between sociodemographic and injury profiles. Age, gender and being unemployed were all found to be significant predictors of acquiring a TSCI. The risk of sustaining a vertebral fracture was found to increase significantly by 0.37 in females and 0.26 in the unemployed respectively. Traumatic SCI was found to be a significant predictor of sustaining a vertebral fracture as well as underdoing spinal surgery. However, a low proportion of those with spinal fractures underwent spinal surgery and were rather managed conservatively. Again, financial and human resource limitations within the public healthcare sector is the likely driver behind this finding. This highlights the need to advocate for resources from theatre time to equipment and specialists. Ultimately, however, preventing TSCI would have a direct economic benefit to the health system [3, 15].

Our study showed a mortality rate of 6.48%. In an acute care unit in the Western Cape, the mortality rate was two-thirds lower at 2.2% [12]. The risk of death in this study increased by 0.92 for every one-unit increase in age. In-hospital mortality rates are reported to be three times higher in low-middle income countries, with mortality being an indicator of quality of hospital care [39]. Madasa et al. [38] reported that almost one-quarter of those sustaining a TSCI died four years after injury. This highlights the need to review acute-care standard operating procedures and address long term follow-up care to maximise health outcomes and limit mortality in this high-risk population.

## Conclusion

Young males are most at risk of SCI and are consequentially admitted for rehabilitation in the public healthcare setting in Gauteng. Assault continues to be the leading cause of SCI in Gauteng, with numbers unrivalled the world over. These injuries tend to be severe, most commonly causing complete paraplegia to the lower thoracic spine. The NTSCI population differed, however, with the older female populations of Gauteng significantly more affected with their injuries most often incomplete in nature. The risk of NTSCI in those with HIV and comorbidities was high, a point of interest for preventative care and an indicator to optimise the ongoing care in these populations. Being unemployed was found to be a risk factor for sustaining a TSCI, a worrying finding given South Africa’s high unemployment rate. Not being married was also found to have a significant association for TSCI. There is an urgent need to realise community and societal reintegration post discharge from rehabilitation while addressing stigma and empowering those with SCI.

## Implications

### Practice

Prevention strategies must be strengthened for both TSCI and NTSCI. While the world implements preventative strategies aimed at transport- and fall-related injuries, South Africa would do well to address the high levels of violence and crime in the fight against TSCI. In the continued absence of an acute public healthcare SCI unit in Gauteng, acute healthcare provision for PWSCI will continue to remain disjointed. It is imperative, then, that continued education and professional development of both the general population and medical personnel working with PWSCI (particularly at tertiary- and regional-level healthcare facilities) is undertaken respectively. Further, patients will continue to wait long periods for their chance in rehabilitation for a stay that generally lasts just short of three months. While the majority of those that are admitted will be deemed fit for discharge by the multidisciplinary team and return to their private residences’, not all will reach this state. While the proportion of those not completing rehabilitation remains less than 10%, as healthcare professionals we should endeavour to realise a successful rehabilitation rate of 100%.

### Research

Surveillance research on SCI is recommended. It is recommended that this same study be conducted at Gauteng’s private healthcare rehabilitation units over the same study period. This will provide grounds for direct comparisons to be made between the two cohorts and give Gauteng a complete picture of SCI rehabilitation. Similar research to that presented here should be conducted in other provinces to provide a more complete picture of SCI in South Africa and help guide policy change by ensuring the best possible care and opportunity for these individual’s post-injury. More research regarding the profile and outcomes of NTSCI is required both locally and abroad.

## Data Availability

The datasets analysed for this study are available from the authors on reasonable request.
